# Meaningful measurements of maneuvers: *People with incomplete spinal cord injury ‘step up’ to the challenges of altered stability requirements*

**DOI:** 10.1186/s12984-021-00840-4

**Published:** 2021-03-02

**Authors:** Wendy L. Ochs, Jane Woodward, Tara Cornwell, Keith E. Gordon

**Affiliations:** 1 Department of Physical Therapy and Human Movement Sciences, Northwestern University, 645 N Michigan Ave, Chicago, IL 60611 USA; 2 Department of Biomedical Engineering, Northwestern University, 2145 Sheridan Rd, Evanston, IL 60208 USA; 3 Edward Hines Jr. VA Hospital, 5000 5th Ave, Hines, IL 60141 USA; 4 Shirley Ryan AbilityLab, 355 E Erie St, Chicago, IL 60611 USA

**Keywords:** Walking, Balance, Spinal cord injury, Maneuvers, Stability, Margin of stability, Force fields

## Abstract

**Background:**

Many people with incomplete spinal cord injury (iSCI) have the ability to maneuver while walking. However, neuromuscular impairments create challenges to maintain stability. How people with iSCI maintain stability during walking maneuvers is poorly understood. Thus, this study compares maneuver performance in varying external conditions between persons with and without iSCI to better understand maneuver stabilization strategies in people with iSCI.

**Methods:**

Participants with and without iSCI walked on a wide treadmill and were prompted to perform lateral maneuvers between bouts of straight walking. Lateral force fields applied to the participants’ center of mass amplified or attenuated the participants’ movements, thereby increasing the capability of the study to capture behavior at varied levels of challenge to stability.

**Results:**

By examining metrics of stability, step width, and center of mass dynamics, distinct strategies emerged following iSCI. The minimum margin of stability (MOS_min_) on each step during maneuvers indicated persons with iSCI generally adapted to amplified and attenuated force fields with increased stability compared to persons without iSCI, particularly using increased step width and reduced center of mass excursion on maneuver initiation. In the amplified field, however, persons with iSCI had a reduced MOS_min_ when terminating a maneuver, likely due to the challenge of the force field opposing the necessary lateral braking. Persons without iSCI were more likely to rely on or oppose the force field when appropriate for movement execution. Compared to persons with iSCI, they reduced their MOS_min_ to initiate maneuvers in the attenuated and amplified fields and increased their MOS_min_ to arrest maneuvers in the amplified field.

**Conclusions:**

The different force fields were successful in identifying relatively subtle strategy differences between persons with and without iSCI. Specifically, persons with iSCI adopted increased step width and reduction in center of mass excursion to increase maneuver stability in the amplified field. The amplified field may provoke practice of stable and efficient initiation and arrest of walking maneuvers. Overall, this work allows better framing of the stability mechanisms used following iSCI to perform walking maneuvers.

## Background

It is difficult to resolve the strategies people use to skillfully stabilize their bodies during walking maneuvers. Stability, the tendency for a system to return to a *consistent* state, is generally considered beneficial during straight walking. However, it has been difficult to quantify stability during maneuvers given that the objective of a maneuver is to safely breach the current state and transition to an alternative stable state (e.g., straight walking in a path parallel but lateral to the previous one). Maneuvering, an essential skill for community ambulation, can be accomplished with varying strategies of foot placement, body movements, and ground-on-foot force control [1]. However, our understanding of how people adapt stepping strategies to manage stability during maneuvers is poor.

The need to understand these stabilizing strategies among people who have sustained a motor-incomplete spinal cord injury (iSCI) is particularly pressing. iSCI disrupts balance and challenges one’s ability to safely and efficiently perform maneuvers. In addition, following iSCI sensory and motor deficits can limit volitional strength, impair coordination, and affect proprioceptive feedback, which may restrict the options for stabilization strategies. Difficulty maneuvering is likely a contributing factor to the reduced mobility [2] and high fall rate [3] observed among ambulatory individuals with iSCI. A greater understanding of how people manage stability during walking maneuvers could provide valuable insight for designing more effective interventions to enhance the ability to maneuver after iSCI.

**Fig. 1:**
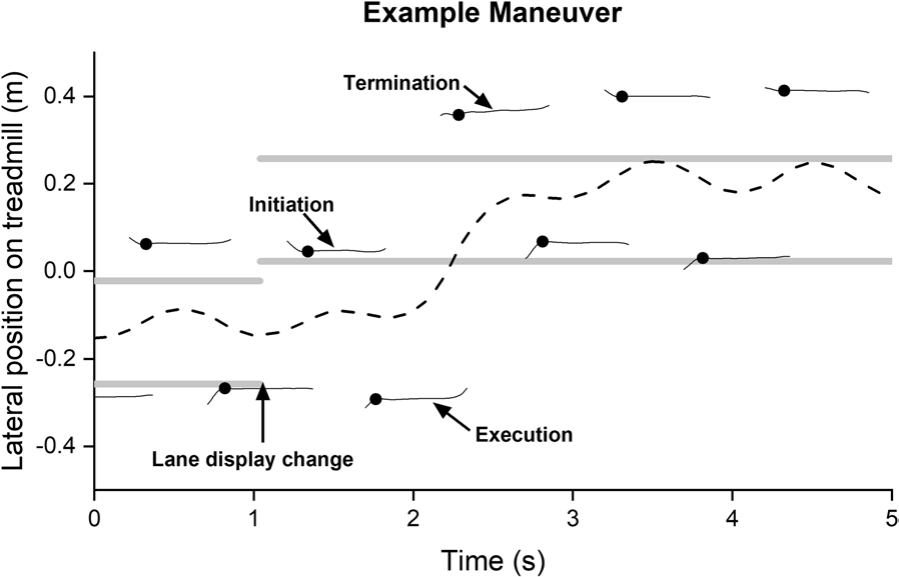
Example data from a lateral maneuver during walking (non-iSCI participant 4, maneuver in Null field). Participants were instructed to keep their COM (dashed line) within a lane projected on the treadmill (thick gray lines). The location of the lane changed during single-leg stance of the limb contralateral to the direction of the next maneuver, cueing the participant to laterally maneuver. The edge of the base of support is shown for each step (thin black lines), with a black dot marking the time that the MOS_min_ occurred. Three steps were analyzed from each maneuver: initiation (the step on the ipsilateral foot following lane location change), execution (the step following initiation), and termination (the step ipsilateral to the maneuver direction during which the COM entered the new target lane).

To better understand how a person performs lateral ‘lane change’ maneuvers during forward walking [4] (Fig. 1), we can apply continuous lateral force through the person’s COM during a maneuver that is proportional to lateral COM velocity. In opposing movement, this is a damping force field denoted *movement attenuating.* A damping force removes energy only when the COM is moving, providing increases and decreases in force that are as gradual as the velocity of the COM increases and decreases. This avoids sharp changes in perturbation that could unnecessarily destabilize the individual. Conversely, force applied with this pattern in the *same* direction as COM velocity is a negative-damping force field, denoted *movement amplifying*. Use of an amplifying field provides consistency in the pattern of force applied to the person but does so in the opposite direction of the attenuating field. Maneuvering requires a lateral velocity–time profile of the COM that is different from straight walking, including a prolonged period of COM excursion in the direction of the maneuver and the subsequent arrest of that motion. Thus, lateral velocity-dependent force fields allow for richer characterization of the maneuver by altering the physical requirements for breaching and then reestablishing forward walking stability. Attenuating lateral COM velocity will increase frontal-plane stability during forward walking, which should resist the transition into a lateral maneuver but assist the arrest of the maneuver. Vice versa, amplifying lateral COM velocity will decrease frontal-plane stability during forward walking [5], which should assist the transition into a lateral maneuver but increase the challenge to arrest the maneuver. The current study introduces both Attenuated and Amplified fields to a lateral maneuver task to evaluate how different stability requirements affect the strategies people with and without iSCI use to maneuver.

Similar attenuating [6] and amplifying [5, 6] force fields have been valuable for understanding stability-related consequences of the stepping strategies adopted during straight walking. People with and without iSCI tend to modify lateral margins of stability (MOS), the distance between a velocity-adjusted COM position and the edge of an individual’s base of support (BOS), in response to changes made by external viscous force fields. By increasing or decreasing lateral MOS, the impulse needed to cause frontal plane instability (based on an inverted pendulum model of walking [7]) can be changed in accord with the challenges of a task. Thus, the adaptive stepping strategies and associated changes in lateral MOS used to first breach and then reestablish forward walking stability for a lateral maneuver are expected to manifest on steps initiating, executing, and terminating lateral maneuvers (Fig. 1) in the presence of force fields that attenuate and amplify lateral COM velocity.

To address gaps in understanding the stability and stepping during maneuvers, this study characterized strategies used by people with and without iSCI performing lateral “lane-change” maneuvers during forward walking in Attenuated, Amplified, and Null force fields. Given the increased challenge of maneuvering compared to straight walking, we expected that participants with iSCI would maintain a larger MOS compared to their peers without iSCI regardless of potential interaction effects with field and step. Additionally, considering findings of maneuvering without force fields [1, 8], we expected the following relations between *steps* regardless of potential interaction effects with group and field: (1) MOS was expected to be smallest on the initiation step (Fig. 1) relative the execution and termination steps as participants bias their COM in the maneuver direction in anticipation of the impending movement. (2) MOS was expected to be largest on the execution step as individuals generate a lateral impulse by pushing off of the limb contralateral to the maneuver direction.

Given the adaptability of stepping behavior in previous work [5, 9], more specific hypotheses were made considering the interaction effects between groups and fields by step. The Attenuated field may be advantageous during the maneuver termination, while the Amplified field may be advantageous during the maneuver initiation/execution. The following hypotheses were made within groups for each step regarding the Attenuated or Amplified fields compared to the Null field. In the Attenuated field, we hypothesized that relative to the Null field, participants would (1) decrease the minimum lateral MOS (MOS_min_) on the initiation step (Fig. 1) to facilitate the maneuver by biasing COM position towards the maneuver direction, (2) increase MOS_min_ on the execution step to increase potential for lateral ground-on-foot force in the direction of the maneuver to counter the opposing field, and (3) decrease MOS_min_ on the termination step to take advantage of the field reducing the need to brake. In the Amplified field, we hypothesized that relative to the Null field, participants would (1) increase MOS_min_ ipsilateral to the maneuver direction on the initiation step to afford increased stability, anticipating the assistance of the force field to overcome that stability on the subsequent execution step (2) decrease the MOS_min_ contralateral to the maneuver on the execution step to leverage assistance from the force field and (3) increase the lateral MOS_min_ ipsilateral to the maneuver direction on the termination step to prevent overshoot of the target end-position. We expected the termination step MOS_min_ increase to be especially evident in individuals with iSCI given their intensified cautious response to destabilizing fields in previous work [6]. Step width, COM excursion, and COM peak velocity were also quantified to further unpack the strategies contributing to differences in MOS_min_.

## Methods

### Participants

A convenience sample of 24 people provided informed consent and participated in the study. Northwestern University and Edward Hines Jr. VA Hospital Institutional Review Boards approved the study protocol. Participants (Table 1) included 12 adults with iSCI (injury level ranging from C3 to T9) and 12 age- (± 5 years) and gender-matched individuals with no documented neurological or balance impairments (iSCI age 48 ± 15 years, non-iSCI age 47 ± 15 years, 4 females in each group). Inclusion criteria included: spinal cord injury level between C1-T10, American Spinal Injury Association Impairment Scale (AIS) C or D, > 6 months since initial injury, range of motion within functional limits of ambulation, ability to walk 10 m without assistive devices or physical assistance, no excessive lower limb spasticity of the quadriceps or hamstring muscle groups as measured by a score of > 3 on the Modified Ashworth Scale, and ability to tolerate 10 min of standing. Exclusion criteria included severe cardiovascular or pulmonary disease, recurrent fracture history, known lower extremity orthopedic problems, concomitant central or peripheral neurologic injury, and inability to provide informed consent due to cognitive impairments.

**Table 1:** Demographics and mobility profiles of the study participants are shown

Participant metrics										
Participant	Sex	Age (years)	Preferred speed (m/s)	FGA	SSV 10MWT (m/s)	FV 10MWT (m/s)	SCI level	WISCI II	L LEM score	R LEM score
iSCI										
S1	M	61	0.8	21	1.0	1.5	C3–C5	18	17	25
S2	M	61	0.7	19	0.9	1.0	C6	20	21	25
S3	M	55	0.4	17	0.6	1.1	C5–C7	20	24	25
S4	M	67	0.5	21	1.0	1.3	C3–C7	20	25	25
S5*	*M*	*35*	*0.9*	*30*	*0.9*	*1.5*	*C7*	*20*	*25*	*24*
S6	M	19	0.9	28	1.2	1.4	C4	20	24	25
S7	M	62	0.7	17	0.8	0.9	C3–C4	20	24	23
S8	M	33	0.4	12	0.5	1.2	T8	18	24	16
S9	M	53	0.9	30	1.2	1.7	C4–C5	20	25	25
S10	F	52	0.5	20	0.7	1.0	C6	20	23	23
S11	M	52	0.4	17	0.7	0.9	C5	18	19	25
S12	F	29	0.3	9	0.3	0.5	T8–T9	20	25	20
Mean ± SD		49.5 ± 15.5	0.6 ± 0.2	19.2 ± 6.1	0.8 ± 0.3	1.1 ± 0.3	Median (Q1–Q3)	20 (19.5–20)	24 (22.5–25)	25 (23–25)
Non-iSCI										
C12	M	56	1.1	30	1.6	2.1				
C9	M	63	1.1	30	1.3	1.8				
C5	M	54	0.8	29	1.1	2.1				
C6	M	71	0.9	29	1.4	2.1				
C10	M	36	1.1	30	1.5	2.2				
C2	M	20	0.8	30	1.3	1.8				
C11	M	64	1.0	28	1.3	1.7				
C8	M	30	1.3	30	1.4	1.9				
C3	M	48	0.9	29	1.1	1.6				
C1	F	51	0.8	27	1.3	2.2				
C7	M	48	1.2	28	1.3	1.9				
C4	F	26	1.2	30	1.8	2.2				
Mean ± SD		47.3 ± 16.1	1 ± 0.2	29.2 ± 1	1.4 ± 0.2	2 ± 0.2				

*Data from participant S5 was excluded from analysis due to an incomplete data set

### Experimental setup

Participants walked on an oversized treadmill (belt width 1.39 m; Tuff Tread, Willis, TX) that provided room to safely perform lateral walking maneuvers. Participants wore a trunk harness attached to an overhead anchor (ZeroG Passive, Aretech). The harness provided no support during walking but could catch the participant in the case of a fall. Attachment of the harness to the overhead anchor was adjusted for each participant so their ability to perform lateral maneuvers was not restricted. Participants did not use handrails, or assistive devices during trials. As an additional safety precaution, spotters provided non-contact guard to participants with iSCI during treadmill walking.

Participants received visual feedback about their lateral position on the treadmill from a projection of a line representing the lateral position of the COM (estimated in real time as the midpoint of greater trochanter motion capture markers) and a target “lane” (Fig. 1, 0. 25 m wide) on the treadmill belt. The lane was offset to the left or right half of the treadmill, depending on the intended maneuver direction. Participants walked at their preferred treadmill speed (iSCI 0.60 ± 0.2 m/s, non-iSCI 1.0 ± 0.2 m/s) and were instructed to do their best to keep their COM line within the lane. To cue maneuvers, the lane projection location on the treadmill was instantaneously moved to the opposite side (right or left) of the treadmill. The distance between lane centers of the prior and new target lanes was 30 cm.

Participants were instructed to maneuver as safely and efficiently as possible to the new lane location. Once the participant’s COM entered the new target lane, a predetermined number of steps was required before another target lane switch. To reduce the possibility that participants would predict the timing of the target lane switch, the number of steps (3–8 steps) occurring between maneuvers was randomized every maneuver and unknown to the participant. The target lane switch always occurred ~ 100 ms after a heel strike of the foot contralateral to the maneuver direction (i.e. right heel strike when the lane was to switch from the right to left side of the treadmill, and vice versa).

Participants performed the walking maneuvers in three lateral force field conditions: Attenuated, Amplified and Null. Participants wore a harness around their hips that was snug but allowed for typical lower-limb motion. Each side of the harness attached to a separate tensioned cable that extended out horizontally in the person’s frontal plane and routed through a series of pulleys to attach to one side of the Agility Trainer robotic device [10]. The robotic device consists of a series elastic actuator powered by a linear motor for each side of the person. Load cells in series with the cables and position-sensing optical encoders within the series elastic actuator are inputs to a nested proportional-derivative controller [10] to continuously produce a commanded force on the participant based on the excursion of the cables. For the current study, the sensed position was used to derive real-time velocity of the approximate COM and produce proportional lateral force with minimal delay (~ 30 ms [10]) on the participant’s pelvis in two of three experimental conditions (Attenuated and Amplified). The system was controlled using a cRIO-9074 FPGA with LabVIEW Real-Time software (National Instruments, Austin, TX). On Attenuated and Amplified Field trials, the appropriate field was turned on in LabVIEW by an experimenter before the treadmill started and turned off when the trial was complete and the treadmill stopped.

In the Attenuated and Amplified conditions, the force was in the opposite and same direction as lateral COM velocity, respectively. These fields used a viscous gain of ± 40 Ns/m in their respective directions, similar in magnitude to fields used in previous studies [5, 11]. Forces were also capped at 80 N for all participants for safety. During the Null condition, the cables were not attached to the harness and thus exerted no force on the participant.

A 12-camera motion capture system (Qualisys AB, Gothenburg, Sweden) recorded 3D marker locations at 100 Hz. Thirteen active-LED motion capture markers (3 markers on pelvis, bilaterally on the greater trochanters, lateral malleoli, calcanei, and second and fifth metatarsals) were used to capture lower-limb kinematics. Force sensing resistors were attached to the bottom of each foot to detect steps in real time with signal transmission via the Delsys Trigno wireless acquisition system (Delsys, Natick, MA).

### Protocol

Demographic measure collection and clinical assessments of strength and walking function were performed by a licensed physical therapist before the experimental protocol. Clinical tests for participants with iSCI included the lower extremity motor score (LEMS) portion of the American Spinal Injury Association Impairment Scale (AIS), the 10 m Walk Test (10MWT [12–14]) performed at each participant’s self-selected (SSV) and fast (FV) velocities, the functional gait assessment (FGA) [15], and walking index for spinal cord injury (WISCI II) [16, 17]. Individuals without iSCI performed the FGA and 10MWT. All participants completed the tests without walking aids. For treadmill walking, participants’ preferred speeds were assessed by iterating changes in treadmill speed until the participants reported their preference (~ 2 min walking). Preferred speed was determined when walking with no external assistance. For the main experiment, participants performed trials in three force field conditions (Null, Attenuated, and Amplified). The order of the 3 force field conditions was randomized for each participant, and all walking was performed at an individual’s preferred speed. During each condition, participants performed the following in order:

1. Maneuver practice in the force fields—treadmill off: Participants performed four lateral maneuvers with the field on and treadmill speed set to zero to gain an initial sense of the task in the field.
2. Maneuver practice in the force fields—treadmill on: Participants performed one minute of straight walking with the field on and the treadmill moving at the participants preferred speed immediately followed by four lateral walking maneuvers with the treadmill on to further familiarize the participant with the task in the field.
3. Rest: 30 s standing rest.
4. Maneuver task in the force fields with the treadmill on: Participants performed eight walking maneuvers in the field with the treadmill moving at their preferred walking speed. Data from this task was used for analysis.

### Processing and analysis

Kinematic marker data was processed using Visual3D (C-Motion, Inc., Germantown, MD). Marker data was gap-filled (3rd order polynomial with maximum gap of 10 frames) and low-pass filtered (Butterworth, 6 Hz cut-off frequency). Mediolateral COM position and velocity were calculated in Visual3D using the built-in “Visual3D pelvis” model and pelvis marker data.

Step metrics were calculated using a custom LabVIEW (National Instruments, Austin, TX) routine. Lateral MOS_min_ [7] during stance period (heel strike to toe-off) was used to assess stability during the maneuver initiation, execution, and termination steps (Fig. 1). MOS was calculated during the stance phase of each foot using the lateral malleoli as the edge of the BOS, and the MOS_min_ identified as the smallest MOS occurring within each step. Step width, COM excursion, and COM peak velocity were also calculated. Step width was calculated as the lateral distance between calcaneus markers at heel strike of the steps assessed for MOS_min_. COM excursion was the lateral distance between the furthest left and right excursions of the COM during stance and peak velocity was the largest lateral speed of the COM toward the stance foot in that period.

To assess differences in MOS_min_, step width, COM excursion, and peak lateral COM velocity among the three maneuver steps (initiation, execution, and termination), two groups (iSCI and non-iSCI), and three fields (Null, Attenuated, and Amplified), a linear mixed-effects model was fit for each metric using maximum likelihood estimations in SPSS (IBM). The models specified fixed effects for group and step and the interactions between step, group, and field with random intercepts for participants. Each maneuver was treated as a single observation, totaling eight observations per step per field per person. When significant effects between steps or step-group-field interactions were identified, pairwise linear contrasts were made to further evaluate significance. All comparisons were evaluated at a significance level of *α* = 0.05 with Bonferroni correction when multiple comparisons were made.

## Results

### Participants and general protocol

Participants with and without iSCI were able to perform the maneuver task in all three fields. One participant with iSCI was excluded in final statistical analysis due to missing motion capture data. Metrics by participant are shown in Table 1.

Across conditions and groups, participants typically followed the pattern of stepping shown in Fig. 1. That is, participants waited almost a full gait cycle after the target lane changed location during mid-stance of a step on the foot contralateral to the maneuver direction to begin movement into the new lane (execution step, Fig. 1). Participants had their COM in the target lane by heel strike of the step following the “termination step” (Fig. 1). For most maneuvers (80.8%), there were no intermediate steps between the execution and termination steps, as illustrated in Fig. 1. A few maneuvers within both groups, particularly in the Attenuated field, had one (17.9%) or at most two (1.2%) intermediate steps on the foot contralateral to the maneuver direction before the COM was in the target lane. The COM path and MOS of an example maneuver are also shown in Fig. 1.

### Effects of group and step on margin of stability

Overall, participants with iSCI had significantly greater MOS_min_ compared to their peers without iSCI (effect of group, p = 0.026). Figures 3, 4 and 5 show the distribution of MOS_min_ measurements across conditions and groups and significant interactions between groups and fields within steps.

**Fig. 2:**
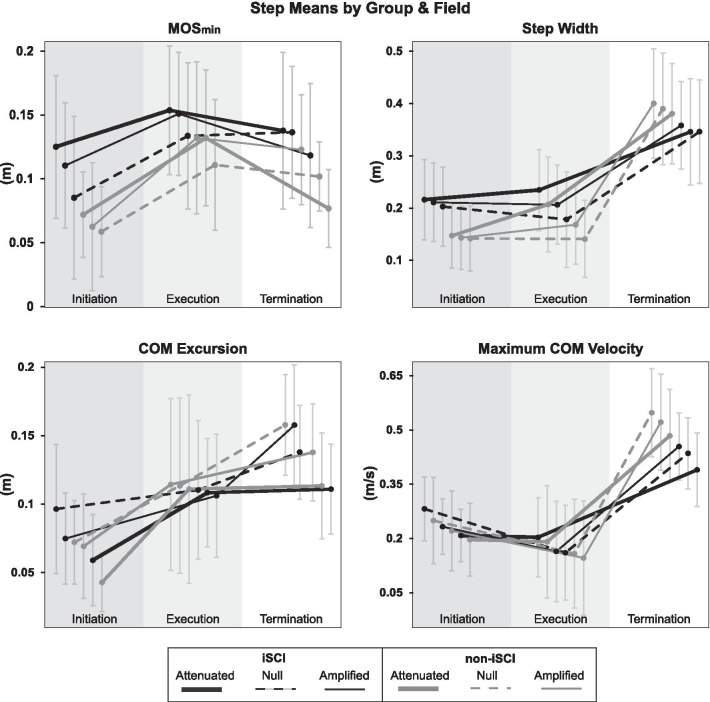
Points with standard deviation bars represent the mean and distribution of outcomes across trials and participants within each category. Trends for step metrics across steps and fields illustrate differences in maneuver strategies between participants with iSCI (points connected by black lines) and without iSCI (points connected by grey lines). Attenuated (thick solid lines), Null (dashed lines) and Amplified (thin solid lines) fields affected the requirements for breaching and regaining stability, further illuminating differences in strategies between groups

MOS_min_ was significantly different between the initiation, execution, and termination steps and consistent with the hypothesis that MOS_min_ would be smallest on the initiation step (initiation < execution, p = 0.000 and initiation < termination, p = 0.000). Interactions specifically yielded p < 0.05 for all between-step comparisons within groups and fields except for the initiation versus termination comparison in the Attenuated and Amplified fields within iSCI. The results also supported the hypothesis of the largest MOS_min_ on the execution step (Execution > Initiation, p = 0.000 and Execution > Termination, p = 0.000). Interactions specifically yielded p < 0.05 for all between-step comparisons within groups and fields except for execution versus termination in the Null field in both groups, and for execution versus termination in the Amplified field in non-iSCI. Figure 2 shows mean trends across steps within groups and fields.

### Interactions by step: Initiation

**Fig. 3:**
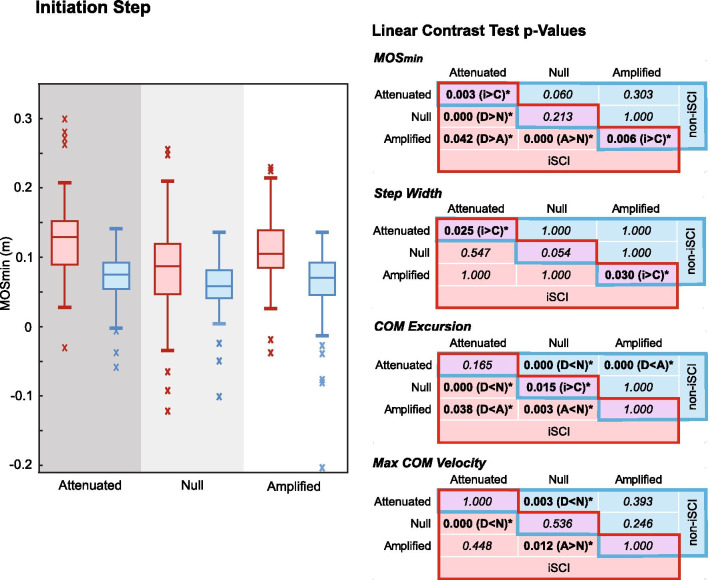
Boxplots showing the medians and distributions of MOS_min_ on the initiation step across trials show similar trends across fields for both persons with iSCI (red, left plots) and without iSCI (blue, right plots). Significant differences between fields within groups (iSCI in red cells on lower left of tables, without iSCI in blue cells on upper right of tables) and between groups within fields (purple cells on diagonals) reveal the significantly different step attributes co-occurring with significant differences in MOS_min_

On the initiation step (Fig. 3), participants with iSCI had a significantly larger MOS_min_ than those without iSCI in both the Attenuated (p = 0.003) and Amplified (p = 0.006) force fields. This occurred with individuals with iSCI exhibiting significantly larger step widths in the Attenuated and Amplified fields and COM excursion in iSCI in the Null field.

Within participants with iSCI, the initiation step MOS_min_ (Fig. 3) was significantly larger in the Attenuated (p = 0.000) and Amplified (p = 0.000) fields compared to the Null field, and in the Attenuated compared to Amplified field (p = 0.042). These larger MOS_mins_ values occurred with significantly less COM excursion than in the Null field despite lower and higher peak COM velocities in the Attenuated and Amplified fields compared to the Null field, respectively.

Within participants without iSCI, there were no significant differences between fields on the initiation step MOS_min_. However, COM excursion was smaller in the Attenuated field compared to the Null and Amplified fields and slower in the Attenuated than Null field.

### Interactions by step: Execution

**Fig. 4:**
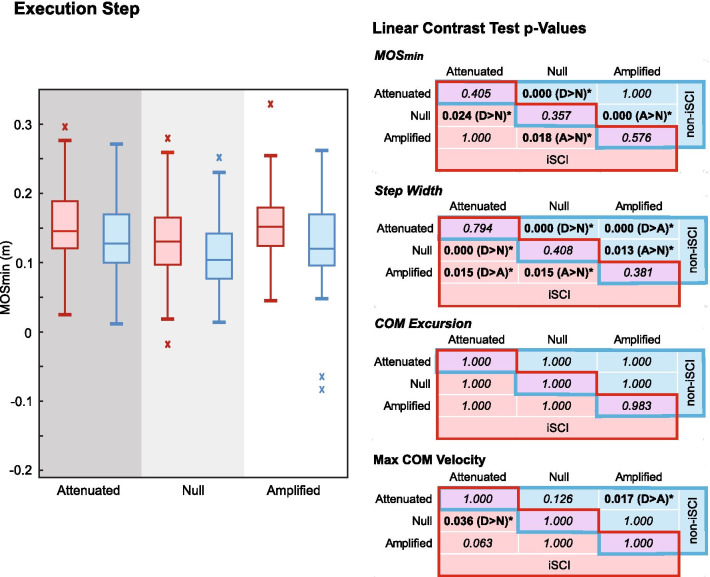
Boxplots showing the medians and distributions of MOS_min_ on the execution step across trials show similar trends across fields for both persons with iSCI (red, left plots) and without iSCI (blue, right plots). Significant differences between fields within groups (iSCI in red cells on lower left of tables, without iSCI in blue cells on upper right of tables) and between groups within fields (purple cells on diagonals) reveal mainly significantly different step width co-occurring with significant differences in MOS_min_

On the execution step (Fig. 4), there were no significant differences in MOS_min_ or stepping metrics between groups. Within participants with iSCI, MOS_min_ was significantly larger in the Attenuated (p = 0.024) and Amplified (p = 0.018) fields compared to the Null field. Step width was different between all fields in the iSCI group (Attenuated > Amplified > Null), and peak COM velocity was greater in the Attenuated than Null field.

Similarly, participants without iSCI had significantly larger MOS_min_ (Fig. 4) in the Attenuated (p = 0.000) and Amplified (p = 0.000) fields compared with the Null field as well as different step widths (Attenuated > Amplified > Null). Individuals without iSCI showed greater peak COM velocity in the Attenuated field compared to the Amplified field.

### Interactions by step: Termination

**Fig. 5:**
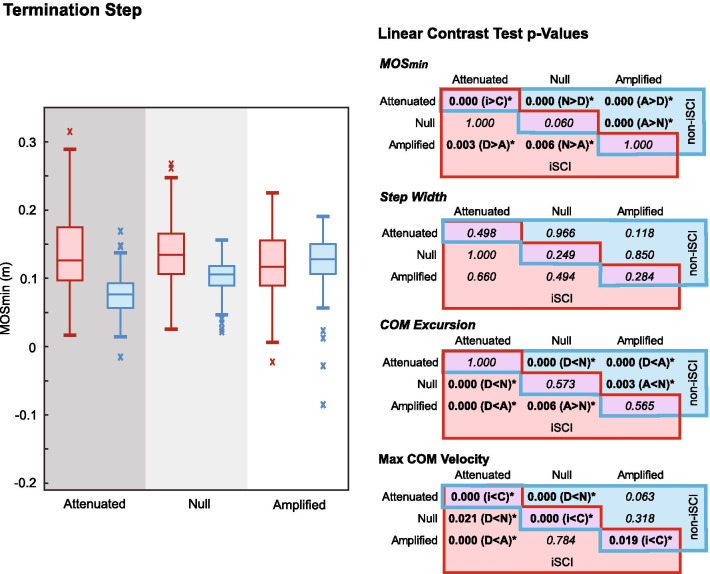
Boxplots showing the medians and distributions of MOS_min_ on the termination step across trials show distinct trends across fields for between persons with iSCI (red, left plots) and without iSCI (blue, right plots). Significant differences between fields within groups (iSCI in red cells on lower left of tables, without iSCI in blue cells on upper right of tables) and between groups within fields (purple cells on diagonals) reveal the significantly different step attributes co-occurring with significant differences in MOS_min_

On the termination step (Fig. 5), participants with iSCI had a larger MOS_min_ than their peers without iSCI in the Attenuated field (p = 0.000) but did not significantly differ in the other fields. Interestingly, there were no significant differences in step width between groups or fields, and thus, the larger termination step MOS_min_ in the Attenuated field is likely attributable to reduced COM excursion and peak COM velocity in persons with iSCI. The adaptation to the Attenuated field appears to largely occur as increased step width on the preceding execution step. People with iSCI tended to step similarly wide on the execution step, but peak COM velocity was smaller across fields compared the participants without iSCI.

Within participants with iSCI, the termination step MOS_min_ was larger in the Attenuated (p = 0.003) and Null (p = 0.006) fields compared to the Amplified field. COM excursion was different between all fields, with the smallest values in the Attenuated field and largest in the Amplified field (Fig. 5). Peak COM velocity was also smaller in the Attenuated field compared to the Null and Amplified fields.

Within participants without iSCI, the termination step MOS_min_ (Fig. 5) was significantly different between all fields, with the smallest in the Attenuated field (p = 0.000 between all fields) and largest in the Amplified field. COM excursion was also different between all fields, but unlike the iSCI group, the smallest excursions were in the Attenuated field and the largest were in the Null field. Peak COM velocity was also smaller in the Attenuated field than Null field.

## Discussion

Maneuvering is an essential component of walking, yet its complexity makes it difficult to characterize and address stability of this behavior, particularly when injury such as iSCI imparts significant coordination and strength deficits. This study investigated the stability and stepping strategies persons with and without iSCI use to laterally maneuver without an external force field and in the presence of Attenuated and Amplified force fields. These fields modified the stability requirements to first transition from forward walking into a lateral maneuver and then arrest the lateral maneuver to resume forward walking.

### Margin of stability between groups

Study of lateral maneuvers in persons with and without iSCI has revealed trade-offs between stability and maneuverability [1, 8], and the current study adds to our understanding of people’s preferences and/or abilities. When laterally maneuvering, individuals must weigh minimizing mechanical energy costs, maintaining stability, and producing adequate lateral ground-on-foot force to maneuver. The effect of group showed a larger MOS_min_ in participants with iSCI compared to those without iSCI as expected (Fig. 2). This between-group difference was dominated by significant interactions with field in the Attenuated and Amplified fields on the initiation step and in the Attenuated field on the termination step. Cautious response to the force fields by persons with iSCI—regardless of force direction—may have emphasized the larger MOS_min_ values that yielded significance compared to their peers without iSCI. While it may be expected for individuals with iSCI to increase cautiousness and therefore MOS_min_ in the Amplified field, the larger MOS_min_ could be surprising in the Attenuated field, as a previous study of straight walking [6] showed adaptation to a smaller MOS in an Attenuated field in persons with and without iSCI. As hypothesized, however, the complexity of maneuvering may have added enough challenge to prompt adaptation of a more cautious strategy with iSCI. Therefore, individuals with iSCI may have elected a larger initiation step MOS_min_ in the force fields out of an abundance of caution, whereas those without iSCI may have taken advantage of the Attenuated field, relying on its stabilizing contribution to maneuver initiation and termination, and permitted the Amplified field to assist in breaching stabilization to initiate the maneuvers.

### Initiation step

Based on previous work on maneuvers without force fields, we expected individuals to have a smaller MOS on the initiation step compared to the execution and termination steps, which may allow for a faster maneuver but introduces stability vulnerability [4, 8]. As anticipated, the MOS_min_ on the initiation step was smallest in nearly all conditions. This behavior likely indicates that reduced stability in anticipation of a maneuver in a known direction was considered an acceptable risk in exchange for enhancing maneuverability upon initiation of the task. Both groups did not have a significantly smaller MOS_min_ on the initiation step compared to the termination step in the Attenuated field, however, but for different reasons. Persons with iSCI had a relatively *larger* MOS_min_ and step width on the *initiation* step (significantly larger in iSCI compared to non-iSCI, as well, Fig. 3), while persons without iSCI had a relatively *smaller* MOS_min_ on the *termination* step. On the initiation step, the presence of a force field with iSCI, though predictable, may have increased cautiousness, whereas those without iSCI may have taken advantage of the Attenuated field by relying on its stabilizing contribution.

Interestingly, the larger MOS_min_ in the Amplified field within the iSCI group occurred with conflicting COM motion. Individuals with iSCI exhibited smaller COM excursions but greater peak COM velocity in the Amplified field than in the Null field. The smaller excursion may have contributed to the larger MOS_min_, but a greater peak COM velocity suggests COM movements may have actually been less controlled. In contrast, in the Null field, participants with iSCI had large COM excursions, which were significantly greater than in persons without iSCI (Fig. 3). This change may account for the significant decrease in excursion seen in the Amplified field. Thus, maneuvering in force fields may be a means for facilitating persons with iSCI to practice maneuvering with smaller COM excursions during the initiation step, which is not only more similar to persons without iSCI, but potentially more stable, safe, and energetically efficient [9].

Contrary to the hypothesis, participants without iSCI did not change their MOS_min_ between fields on the initiation step. Given individuals’ capacity for generating corrective lateral impulses, the fields may have been perceived as manageable without any advantage gained though modulation of MOS_min_.

### Execution step

On the execution step, both groups utilized greater MOS_min_ and wider steps in the non-zero fields compared to the Null field (Fig. 4). Despite the increase in energetic cost [1], it was hypothesized that the execution step in the Attenuated field would have wide side-stepping that can yield a large MOS and minimize the disturbance to frontal plane angular momentum associated with large lateral ground-on-foot force (such as that needed to maneuver against an Attenuated field). The opposite was hypothesized for the Amplified field, however, where the field acts in the direction of the maneuver (i.e. assisting it) so a smaller lateral ground-on-foot force magnitude is needed in the direction of the maneuver. The increases in execution step MOS_min_ and step width in the Amplified field may have been a method to oppose any excess movements emphasized by the field (possibly in anticipation of the braking necessary on the subsequent termination step), or failure to take advantage of the excursion assistance. This stabilizing behavior, surprisingly, did not occur more markedly in persons with iSCI. The similarity between groups may have been due to the relative novelty of the amplifying field; providing further practice in subsequent studies may better reveal differences on the execution step if ability limits persons with iSCI to such a stabilizing strategy but not those without iSCI.

### Termination step

On the termination step, the contribution of the force fields to the maneuver task reverses. That is, the Attenuated field assists in lateral braking (lateral ground-foot force opposite in direction to the maneuver), while the Amplified field opposes and necessitates more self-produced braking. This motivated the hypothesis that the termination step MOS_min_ would be smaller in the Attenuated field than in the Null, however, experimentally MOS_min_ in the Attenuated field was not significantly different from Null for those with iSCI (Fig. 5). For both populations, COM excursion and peak velocity were smaller in the Attenuated field compared to Null field, indicating the larger MOS_min_ on the termination step was likely a consequence of the field.

With the proposed challenge to maneuver termination, it was hypothesized termination step MOS_min_ would be greater in the Amplified field than in the Null. The termination step MOS_min_ in the Amplified field showed different relative behavior in each group. Participants in both groups had similar MOS_min_ in the Amplified field; however, relative to the Null field, those without iSCI had a larger MOS_min_ while participants with iSCI had a smaller MOS_min_ (Fig. 5). Individuals without neurological injury behaved consistently with the hypothesis that a larger MOS_min_ would be used to avoid target overshoot in the destabilizing field. The larger MOS_min_ occurred with similar step width but reduced COM excursion, demonstrating what seems to be a controlled maneuver without the need for adapted foot placement. The opposite was seen in those with iSCI, where the reduced MOS_min_ occurred with greater COM excursion and, again, consistent foot placement. Assuming that individuals with iSCI would have reduced COM excursion like those without iSCI if they were capable, this difference between the groups highlights maneuver termination in the Amplified field as a particularly challenging task. Although specific strength and/or coordination deficits are not clear, the reduced stability apparent with iSCI in a task where their peers without iSCI tend to *increase* stability suggests a deficit that may be addressable with practice in such an environment.

### Meaningful maneuver measurements and limitations

The ability of the current study to successfully differentiate behaviors between force field conditions and groups provides valuable perspectives on the study of walking maneuvers in general. Key factors in creating maneuvers that could be compared across groups and repetitions were the constraints placed on the maneuver task. At the risk of becoming non-representative of maneuvers during natural ambulation, carefully chosen control of protocol factors was necessary. This study included relatively comparable repetitions of the task by cueing maneuvers of specific, predictable direction and magnitude with relatively uncertain timing, although maneuvers were cued at a consistent phase of the gait cycle. This attribute successfully prevented the use of cross-over steps, which would have been considered incompatible for comparison within this study. Despite the significant difference in preferred walking speed between groups (Table 1, p = 0.000), continuity of the maneuver task itself across participants was evidenced by the many non-significant between-group comparisons of COM excursion and COM peak velocity across fields and steps.

The differences and similarities observed between persons with iSCI and their peers without iSCI highlight potential areas for further focused intervention and study. Specifically, this work demonstrated value in the use of a maneuver task with different force fields to expose behavioral differences, although it is difficult to ascertain whether the observed behaviors in the current study more strongly reflect personal preferences or boundaries in abilities. The instruction on urgency with which to complete the maneuver (in this study, “as safely and efficiently as possible”) likely provided the most latitude for personal interpretation and preference in behavior. This was particularly evident among persons without iSCI in the Attenuated field, where it was assumed all participants were capable of performing the task without multiple intermediate steps but in some cases, took more than one to reach the target lane. Given the motivation this study provides for the maneuver paradigm as a microscope for understanding stepping strategies, further study manipulating the urgency or number of intermediate steps with which participants perform maneuvers would unpack important questions of ability and preferences that were beyond the scope of the current study. Use of consistent fields and maneuver targets across subjects was considered appropriate for the design of our study given its main objective of characterizing within-subject behavior between fields. Alternatively constrained maneuver paradigms, such as one using force fields and/or target lanes scaled to height or strength, may be better suited for investigations unpacking the heterogeneity between participants, such as specific effects of injury extent and location.

The heterogeneity in the iSCI population is a limiting factor in the current study. We recognize that our participants may have a wide range of clinical presentations due to the nature of their injuries, however, in our participant samples we found that the variance in preferred treadmill walking speed in individuals with iSCI was similar to the group without iSCI (σ^2^_iSCI_ = 0.04 (m/s)^2^, σ^2^_non-iSCI_ = 0.03 (m/s)^2^). As noted above, more constrained maneuver tasks designed to delineate specific injury types is warranted to better understand how the strategies observed in this study are achieved. For example, influence of trunk and arm controllability is variable among ambulatory people with iSCI and stands as a subsequent topic of investigation. In the current study, lateral cables extending from the participants’ hips to the Agility Trainer’s actuators inherently suppressed arm swing, which was helpful for task continuity across participants but also limited translation of findings to walking with unimpeded arm use. While the influence of iSCI on maneuvering likely differed between the participants in our sample, this study nonetheless demonstrated commonalities in their stepping strategies for adjusting to different levels of stability challenge.

## Conclusions

This study used lateral force fields during a walking maneuver task to better understand how persons with and without iSCI adapt their stepping strategies under varied stability conditions. In addition to generally characterizing the strategies used by each group, it provides insight on how to potentially provide persons with iSCI practice that may improve the safety and stability of their maneuvers. The amplified force field, that pushed people in the direction they were already moving, resulted in persons with iSCI using a significantly larger MOS_min_ than persons without iSCI to initiate the maneuver. Persons with iSCI used a larger step width that increased their MOS_min_, but their resultant COM excursion was actually similar to persons without iSCI—a behavior that may be more appropriate for maneuvering without excessive COM excursion. In addition, persons with iSCI were capable of but challenged by terminating maneuvers in the amplified field, as evidenced by their *decreased* MOS_min_ on this step versus the *increased* MOS_min_ on the same step in persons without iSCI. Thus, practicing maneuvers in an amplified field may be valuable as an intervention aimed at improving COM excursion control and maneuver termination ability in persons with iSCI.

## Data Availability

The dataset generated and analyzed during the current study is available in the ARCH Northwestern University Institutional Repository, https://doi.org/10.21985/n2-x1kd-xx86.

## Abbreviations

10MWT: Ten-meter walk test
AIS: American Spinal Injury Association Impairment Scale
BOS: Base of support
COM: Center of mass
FGA: Functional gait assessment
FV: Fast velocity trial of 10MWT
iSCI: Incomplete spinal cord injury
LEMS: Lower extremity motor score
MOS: Margin of stability
MOS_min_: Minimum margin of stability
SSV: Self-selected velocity trial of 10MWT
WISCI II: Walking index for spinal cord injury
